# Detecting and measuring of GPCR signaling – comparison of human induced pluripotent stem cells and immortal cell lines

**DOI:** 10.3389/fendo.2023.1179600

**Published:** 2023-05-24

**Authors:** Gaoxian Chen, Detlef Obal

**Affiliations:** ^1^ Department of Anesthesiology, Perioperative, and Pain Medicine, Stanford University, Stanford, CA, United States; ^2^ Stanford Cardiovascular Institute, Stanford University, Stanford, CA, United States

**Keywords:** GPCR (G protein coupled receptors), signaling transduction, HiPSCs, FRET – fluorescence resonance energy transfer, BRET - bioluminescence resonance energy transfer

## Abstract

G protein-coupled receptors (GPCRs) are a large family of transmembrane proteins that play a major role in many physiological processes, and thus GPCR-targeted drug development has been widely promoted. Although research findings generated in immortal cell lines have contributed to the advancement of the GPCR field, the homogenous genetic backgrounds, and the overexpression of GPCRs in these cell lines make it difficult to correlate the results with clinical patients. Human induced pluripotent stem cells (hiPSCs) have the potential to overcome these limitations, because they contain patient specific genetic information and can differentiate into numerous cell types. To detect GPCRs in hiPSCs, highly selective labeling and sensitive imaging techniques are required. This review summarizes existing resonance energy transfer and protein complementation assay technologies, as well as existing and new labeling methods. The difficulties of extending existing detection methods to hiPSCs are discussed, as well as the potential of hiPSCs to expand GPCR research towards personalized medicine.

## Introduction

G protein-coupled receptors (GPCRs) represent the largest family of transmembrane proteins (1) that translate extracellular signals into intracellular biochemical reactions to mediate basic physiological processes (2). GPCR-targeted drug development has been promoted worldwide, and GPCR-targeted drugs account for more than 30% of the medications approved by the US Food and Drug Administration (FDA) (3). Progress made in GPCR research has provided new insights into GPCR oligomerization (4), biased signaling (5), and allosteric regulation (6), and, interestingly, is mainly based on findings generated in immortalized cell lines, such as human embryonic kidney-293 (HEK293) (7) or Chinese hamster ovary (CHO) cells (8). The homogenous genetic background of these cell lines, combined with an uncontrolled overexpression of target proteins (9), impedes the transfer of the knowledge gained to human cells with individual-specific genetic information. Furthermore, immortal cell lines represent one specific cell type, limiting the transfer of research findings to different cell types. The emergence of human induced pluripotent stem cell (hiPSC) technology may overcome these limitations. HiPSCs can be generated from different somatic cells (i.e., fibroblasts, peripheral blood mononucleated cells) by transfecting four transcription factors (10). Importantly, patient-specific genetic information are maintained in hiPSCs (11), which contributes to their tremendous value in personalized drug discovery (11) while simultaneously avoiding ethical issues raised by conducting embryonic stem cell research.

To gain insight into the canonical signaling pathway response *in vivo*, it is necessary to maintain the expression of each effector in the GPCR signal pathway (11). Therefore, the study of GPCRs in hiPSCs requires advanced labeling and detection methods to overcome the low levels of GPCR expression, allowing for the study of these receptors at their natural expression levels. This necessitates high labeling and imaging efficiency to cope with relatively low levels GPCRs (12). Further, GPCR signaling is a dynamic process. Over the past decades, crystal structure analysis has been widely employed to study GPCR pharmacology (6, 13, 14); however, the static nature limits its ability to investigate dynamic intracellular processes. Thus, single-molecule tracking and protein-protein interaction detection techniques might help investigate the dynamic aspects of GPCR signaling (15). In this review, we will discuss assays for protein interaction analysis, such as resonance energy transfer (RET) and protein complementation assays (PCA), as well as detection modalities for intracellular components (including cAMP and activated G proteins), conformational changes in GPCRs and their downstream effectors, and bimolecular or multimolecular interactions of GPCR with other cellular components. We will also discuss labeling and optical acquisition methods. This review will mainly focus on techniques and methods using hiPSCs as a cellular platform to detect patient specific GPCR signaling effects. While hiPSCs still present a challenge, they are poised to become an integral part of GPCR research and may pave the way for the development of personalized GPCR-targeted drugs due to the enormous advances made in labeling and detection techniques.

## Detection methods

Successful imaging of GPCRs within any living cell relies on the quality of the fluorophores and sophisticated fluorescence detectors (16). Understanding the functionality and limitations of these instruments is a fundamental first step to successfully studying GPCRs in living cells. We will start by describing sensor and microscope requirements commonly used and suitable for GPCR and G protein signaling studies.

### Sensor requirements

Fluorescence imaging and luminescence measurement are two widely used techniques for visualizing and quantifying biological processes. Fluorescence imaging is commonly used for visualizing cellular structures and processes in living cells and tissues, while luminescence measurement is used for quantitative analysis of biological molecules and reactions. Fluorescence imaging provides spatially resolved information and monitors changes in fluorescence intensity over time to gain insight into dynamic cellular processes, while luminescence measurement does not require excitation light, is more sensitive, but typically does not allow visualizing cellular structures or processes.

Fluorescence imaging necessitates the use of highly sensitive sensors to detect generally weak signals without risking fluorescence bleaching from excessive excitation light intensity (17). In contrast, luminescent measurement is produced either chemically (i.e., chemiluminescence) or enzymatically (i.e., biological luminescence) and is largely dependent on the sensitivity of the sensor rather than the excitation light intensity. Two types of sensors have been most prominent in the GPCR field depending on their application: (1) Field imaging sensors and (2) Complementary metal-oxide semiconductor sensors. Field imaging sensors include charge-coupled devices (CCDs) and electron-multiplying CCDs (EMCCDs). EMCCDs boast a high quantum efficiency of around 90% and offer on-chip amplification, thereby enabling them to detect single photons (18, 19), as seen in various GPCR imaging studies (20). Complementary metal-oxide semiconductor sensors (CMOS) have higher frame rates and larger imaging areas when compared to CCDs, but their relatively low quantum efficiency (~60%) has prevented their use in GPCR imaging. Recent advancements, however, such as back-illuminated features that direct photons onto the light-receiving surface, have resulted in CMOS with quantum efficiencies akin to EMCCDs (21, 22) [e.g., 95% for Prime 95B backlit CMOS (23)], making them suitable for GPCR imaging. The other type of single-point detectors, photo-multiplier tubes (PMT), are highly sensitive, low-noise, and rapid-response detectors that are frequently used in scanning methods for GPCRs imaging (24).

In addition to the instrument sensor type, distinct instrument configurations (i.e., specific objectives, filters, motorized filter wheels, and light sources) are required for each imaging modality. For example, total internal reflection fluorescence microscopes (TIRF) utilize objectives with a high numerical aperture (>1.45) (25). Instruments utilized for fluorescence resonance energy transfer (FRET) assays require specific two-way color optical filters for the separation of emission lights. Instruments for conducting bioluminescence resonance energy transfer (BRET) require an electric filter wheel and an intense light source to find the focal plane. To summarize, in order to better achieve FRET and BRET, the choice of imaging sensor and instrument is essential for accurately imaging GPCRs and their functions in live cells. FRET and BRET are the most direct methods for observing protein interactions and can be used to observe live cells, which is essential in exploring GPCR mediated signal transduction pathways and understanding the structure and function of GPCRs (16).

#### Wide-field and confocal microscopy

Various microscopy techniques are utilized in GPCR research to enable fluorescence imaging and FRET-based methods (**Table 1**) (36, 37). These instruments vary in their specific excitation light sources, neutral density filters to regulate exposure to laser light, specific filter sets to separate excitation and emission lights, and detection sensors to convert photons into images (51). As the emission signals originate from either above or below the focal plane area, wide-field microscopy results in reduced contrast and degraded images. Nevertheless, this technique has been used to simultaneously observe hundreds of single molecules (52). In contrast, confocal microscopes control the position and size of the imaging area and the high numerical aperture objectives result in a diffraction-limited focus, allowing the generation of two-dimensional images and three-dimensional stacks with a high signal-to-noise ratio in a short period of time. Confocal microscopy has been used to determine co-localization of GPCR, and its effectors (32) and to track biological processes such as A-family GPCR oligomer biosynthesis in live cells (53). Confocal imaging can also be used for quantification of GPCRs, such as measurements on the stoichiometry of the cerulean tagged-β_2_ adrenergic receptor (AR) and Venus-mini G (engineered GTPase domains of G_α_ subunits) (54). In addition to reducing light contamination outside the focal plane by confocal methods, spectral unmixing analysis (e.g., Ex/Em unmixing) can be used to address donor-acceptor emission light crosstalk to further improve FRET or BRET signals (55).

**Table 1:** Microscope models used in GPCR signaling and spatial distribution.

Imaging mode	Model	Reference
Single-molecular track	Leica AM TIRF	(26)
	Nikon TI-Eclipse inverted microscope	(27)
Fluorescence imaging	Leica TCS SP2	(28)
	Leica TCS SP5	(29)
	Olympus IX81	(30)
	Nikon SMZ1500-HR	(31)
	Zeiss LSM 510	(32, 33)
	Zeiss LSM 710	(34)
	Zeiss LSM 800	(35)
	Zeiss LSM 900	(36–38)
	Zeiss LSM 980	(39)
BiFC	PerkinElmer UltraVIEW VOX Confocal system	(40)
dSTORM^*^	Nikon Eclipse Ti2 inverted microscope	(41)
FRET	Nikon Eclipse Ti2 inverted microscope	(42)
	Zeiss Axiovert 135 inverted microscope	(43)
	Zeiss Axiovert 200 inverted microscope	(44–46)
FLIM-FRET	Nikon, A1 confocal laser-scanning microscope	(47)
BRET	Nikon Eclipse Ti-U	(17, 48)
	Olympus LV200 luminescent microscope	(16)
Bioluminescence imaging	Olympus IX-71 epi-fluorescence microscope	(49)
	Olympus LV200 wide field inverted microscope	(50)

^*^Direct stochastic optical reconstruction microscopy.

#### Total internal reflection fluorescence

In TIRF microscopy, an evanescent light wave is employed to illuminate the sample near the coverslip, allowing for the study of molecules that are located near the surface of the sample. TIRF microscopy is a technique widely used to observe single-molecule fluorescence signals by illuminating extremely thin surfaces of samples (typically between 50-200 nm) (56) and therefore reducing background noise (57, 58). TIRF microscopy is used to observe dynamics of cell membrane components, including single molecules, protein-lipid interactions, membrane trafficking, and the spatial distribution of GPCRs. Additionally, TIRF has been used to visualize GPCR oligomerization (26, 59) and to track the interaction of GPCR with G proteins or β-arrestin (60).

Recently, duo-color TIRF imaging has been used to monitor the diffusion, interaction, and signaling of individual GPCRs or G proteins in living cells (27, 61). These studies revealed that GPCRs switch rapidly between fast and slow diffusion phases and form nanodomains within the plasma membrane (27). In conjunction with sophisticated tracking algorithms, these data demonstrated that the structural or functional conditions within the cell membrane likely govern the non-stochastic movements of GPCRs.

#### Plate reader

Plate readers vary in terms of their detectors and associated optical systems, which affect the detection of low light intensity, high throughput capability, and spectral overlap interference, and are an important component of the methods introduced later. **Table 2** provides a summary of common plate reader models and their primary features. In general, after excitation with a light of a defined wavelength, photon signals are measured in a plate reader by a detector, usually a PMT, and subsequently converted into electrical energy. Light emission from the samples is filtered using a monochromator or reader specific filter system. The configuration of the optical system is critical to increase the signal-to-noise ratio of BRET or FRET, as it determines both the excitation intensity of the donor and the light emitted from the acceptor (75). For example, the choice of filter is critical when using a typical combination of RLuc, GFP, and Coelenterazine h because the emission spectrum of RLuc is particularly broad and has a large overlap with the emission spectra of of enhanced green fluorescent protein (eGFP) and yellow fluorescent protein (YFP) at longer wavelengths. To overcome this problem, donor emission filters can be filtered to transmit shorter wavelengths and block light at the emission wavelengths of YFP or eGFP (75). Measurements should be performed between 0.5 and 5 s per well, with longer test times yielding higher signal intensities, but shorter test times enhancing throughput and reducing time interval related errors. For precise wavelength separations, monochromators are an alternative to filters. A monochromator can precisely separate different wavelengths of light, albeit at the expense diminished intensity. It functions by passing light through a prism or diffraction grating, followed by a slit or pinhole, which separates the light into the expected wavelengths. This allows the user to transmit only a single wavelength of light through the monochromator. During excitation, the light is guided through a narrow slit, a series of mirrors, and diffraction gratings, and then through a second narrow slit before reaching the sample to ensure that the desired wavelength is selected to excite the fluorophore (76). Once the fluorophore is excited, it emits light of a different and longer wavelength, and that emission is captured by another series of mirrors, gratings, and slits to limit the emission to the desired wavelength, which then enters the detector for signal readout. However, monochromators, with their complex mechanics, result in higher costs and are accompanied by significant light loss, requiring higher power excitation light sources than filters. For BRET-only assays, however, CCD-based plate readers can simultaneously image the entire assay plate, which increases the assay speed and throughput. Cho and colleagues have successfully measured NanoBRET utilizing CCD-based plate readers such as ViewLux and FLIPR (77).

**Table 2:** Plate reader models used in GPCR signaling and spatial distribution.

Company	Model	Wavelength Ex/Em*	Reading speed	Light source	Sensor	Appl.	Refs
Berthold Technologies	Mithras LB940	Low-noise photomultiplier tube: 340 – 800nm; photo diode: 200 - 1000nm Ex: 355 nm, 485 nm; 405 nm, 450 nm, 490 nm, 620 nm (with Absorbance option) Em: 460 nm, 535 nm; 400 nm, 515 nm, 480 nm, 530 nm (with BRET option); 615 nm (TRF models) 615, 620, 665 nm (TR-FRET models) 568 nm (AlphaScreenTM option)		Halogen lamp, 75 W (340-700 nm); Xenon flash (TRF models); LASER 680 nm (AlphaScreenTM option)	PMT	BRET	(54, 62)
	TriStar2 LB 942	Ex: 340–700 nm; Em: 380–650nm		Halogen lamp	Low-noise PMT	BRET	(63)
BioTek/Agilent	Synergy H1	Ex: 250-900 nm; Em: 250-700nm Bandpass<=18nm	delay after plate movement=100 ms		PMT	BRET	(64)
	Synergy H4	Ex: 250-900 nm; Em: 250-900nm	96w: 11 s 384w: 22 s 1536w: 43 s	Xenon flash	Red shifted and Low Noise PMT	FRET	(65)
	Synergy Neo	Ex: 280-850nm; Em: 200-850nm	96w: 30 s 384w: 110 s 1536w: 440 s	Xenon flash	Single or dual PMTs	BRET Luminescence	(12, 66)
	Synergy Neo2	Ex: 200-850nm; Em: 200-850nm		Halogen lamp; Xenon flash (TRF module)	PMT	FRET (HTRF)	(41, 42, 67)
BMG Labtech	PHERAstar FS	Ex: 230n-750nm; Em: 230-750nm	384w (#flashes): *1:* 14s, *10:* 38s, 50: 1min 29s 1536w (# flashes): 1: 27s, 10: 1min 52s, 50: 5min 18s	Xenon flash	PMT	DRET; BRET; FRET; (TR-FRET)	(5, 50, 68)
	CLARIOstar	Spectral range: 240-740nm or 240-900nm (red shifted PMT)	96w: 9s 384w: 14s 1536w: 27s	Xenon flash	CCD spectrometer	BRET	(59, 69, 70)
	LUMIstar Omega	Spectral range: 240-740nm	96w: 16s 384w: 47s	Xenon flash	Side window PMT	BRET	(69)
	PHERAstar FSX	Spectral range: 220-1000nm; Em: 230-900nm	384w: 1flash: 14s 1536w: 27s	Xenon flash	Dual PMTs CCD spectrometer	DERET; BRET	(42)
	POLARstar Omega	Ex: 240-740nm; Em: 240-740nm	Full spectrum captured in < 1 s/well for absorbance detection	Xenon flash	Side window PMT	BRET	(41, 71)
Molecular Devices	Flexstation 3	Ex: 250-850nm; Em: 360-850nm	1w: about 50ms 8w: about 1.0s	Xenon flash	Dual PMT	BRET	(72)
	SpectraMax L	Spectral range 380-630nm			Dual PMT	BiLC (NanoBiT)	(9)
PerkinElmer	EnVision	Ex: 230-1100nm; Em: 240-850nm	96w: 37s 384w: 1 m 22s 1536w: 4m 15s	UV Xenon flash	PMT	BRET	(73)
Tecan	Spark	Ex: 230-900nm; Em: 280-900nm	96w (1 1flash:< 14s 384w 1 flash: < 30s; Fast Scan mode, 200-1000 nm, 1 nm steps: < 5 s	Xenon flash	PMT	BRET	(12)
	Infinite 200 Pro	Spectral range: 230-1000nm Ex: 230–850nm; Em: 280–850nm	96w: 20s 384w: 30s	UV Xenon flash	PMT	FRET	(74)

* Ex/Em: excitation and emission wavelength; 96w: 96-well plate; 384w: 384-well plate; 1536w: 1536-well plate.

Given that fluorescence signal measurements are an essential primary method for GPCR imaging studies, the next section will describe the various modalities that have been used to detect GPCR function and signaling using fluorescence signals.

### Resonance energy transfer

**Figure 1:**
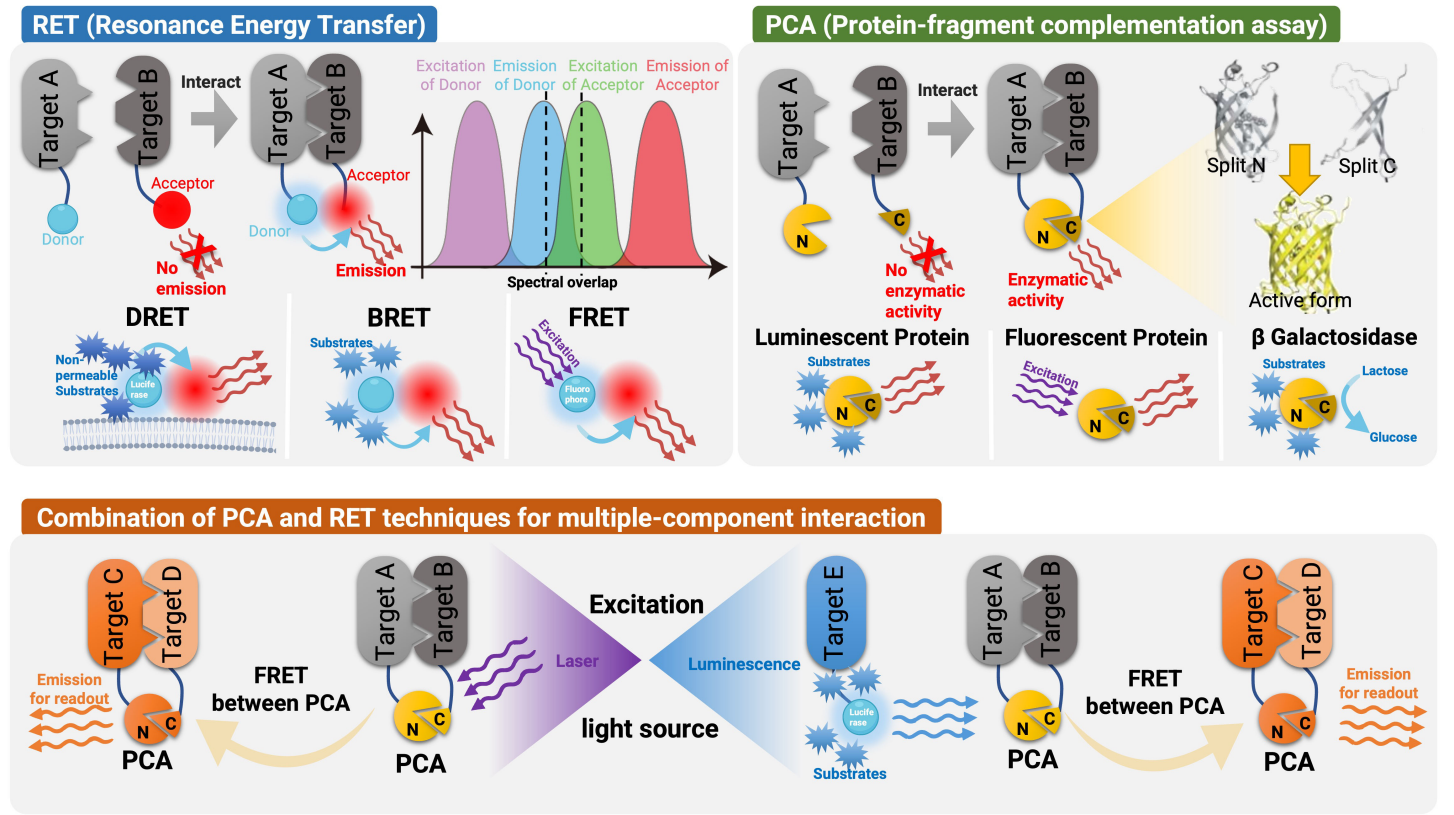
Schematic representation of the detection techniques used in GPCR signaling and spatial distribution. RET is an optical process where energy is transferred from an excited donor molecule to an acceptor molecule *via* dipole-dipole coupling. FRET and BRET are two types of RET phenomena that are used to monitor biological processes. FRET utilizes fluorescent molecules while BRET utilizes bioluminescent molecules and usually has a spectral overlap. DERET is a special form of BRET that characterizes endocytosis of cell surface receptors, in which cell-impermeable substrates are used and their uptake is measured by monitoring the disappearance of donor luminescence. In PCA assays, a reporter protein is split into inactive fragments, which are then fused with potential interacting proteins. When these fragments come into proximity, they spontaneously reassemble into an active luminescent protein, fluorescent protein or β-galactosidase. The combination of PCA and RET has also been applied to illustrate the interaction between multiple components. An external laser source or another Luminescent protein can be used to excite the FRET, allowing detection of interactions among up to five targets.

RET phenomenon, also known as Förster Resonance Energy Transfer or Electronic Energy Transfer is an optical process involving the transfer of energy from an excited molecule (donor) to an acceptor molecule (78). This process is facilitated by dipole-dipole coupling between molecules, involving two types of fundamental particles: electrons and photons. FRET and BRET, which utilize fluorescent and luminescent donors, respectively, have been used to monitor biological processes. For RET pairs, efficient transfer occurs only when the distance between donor and acceptor is below 10 nm, and the transfer efficiency decreases with the sixth power of the intermolecular distance. This is especially relevant considering the average size of a protein is about 5 nm. In addition to the distance between donor and acceptor, the relative orientation of the dipoles is also crucial for efficient energy transfer between RET pairs. RET changes between donor and acceptor fused to the proteins of interest can be used as an indication of the distance between proteins, thereby allowing the characterization of protein-protein interactions and conformation changes within proteins or protein complexes. While FRET assays use fluorescent molecules, BRET assays are based on bioluminescent molecules, characterized by a lower degree of spectral overlap and energy transfer efficiency. Another key advantage of FRET is its ability to provide single-cell resolution, allowing for the study of heterogeneity and variability within a population of cells. This is particularly important in the context of complex biological processes, where individual cells may exhibit different signaling dynamics and responses. The following section will elaborate on the difference between techniques (**Figure 1**).

#### Bioluminescence resonance energy transfer

BRET is a powerful technique for investigating dynamic GPCR signaling in living cells. This technique enables the detection of changes in GPCR conformational states through the fusion of a donor luminescent enzyme and an acceptor fluorescent protein (28, 63), GPCR activity (38), and GPCR regulation mechanisms (48). Four major BRET types (79) are currently used for GPCR research, although the field is rapidly expanding (80). *BRET1* uses coelenterazine h as a substrate and enhanced yellow fluorescent protein (eYFP) as an acceptor with an emission peak of 530 nm. *BRET2*, which has a distance of over 100 nm between donor (RLuc/DeepBlueC) and acceptor (GFP) emission peaks, has a higher signal-to-noise ratio (81) but lower quantum efficiency (17) than BRET1. To compensate for this, RLuc mutations, such as RLuc8 and RLuc-M, have been introduced to increase sensitivity, and the use of the bisdeoxycoelenterazine analogues CLZ400 analogues has extended the measurement time of the assay up to 6 hours (82). *EbBRET*, which utilizes EnduRen™, has a 10-fold higher signal-to-background ratio and can be used in combination with highly sensitive microplate readers to monitor protein-protein interactions for several hours (66). *BRET3* was proposed for enhanced luminescence efficiency with an acceptor-donor spectral interval of up to 85 nm and a peak light output shifted to 564 nm, permitting deeper penetration (16).. NanoBRET is another technique that employs NanoLuciferase as the BRET donor and a range of energy acceptors (83), including fluorescent proteins (84) and the HaloTag dye (85), to investigate molecular interactions in living cells. The use of long-lived substrates like furimazine, vivazine, and endurazine enables measurements ranging from 2 to 48 hours.

BRET does not require excitation light, resulting in lower background and avoiding auto-fluorescence interference; moreover, it does not induce photo-bleaching, leading to fewer damaged cells. However, a paired substrate is required, which can be costly if used in massive quantities, necessitating economic considerations. BRET has been used to develop luminescence-based biosensors for monitoring the downstream effects of GPCRs, including Ca^2+^, cAMP, ERK activity, ATP concentration, membrane voltage, and ubiquitination (49, 65, 71, 86, 87). In addition, BRET assays have been utilized to study GPCR activation, the assembly of GPCR signaling elements on endosomal surfaces, and the conformational dynamics of the receptor-G protein complex; however, the intensity of these sensors has been suboptimal. To address this issue, researchers developed the ‘Nano-lantern’ (88), a protein consisting of an optimized RLuc directly bound to an enhanced YFP variant, that is ten times brighter than the RLuc alone. In addition, the development of effector to plasma membrane translocation (EMTA) has broadened the application of BRET (12, 48). EMTA does not require modifications of receptors or G proteins, making it suitable for primary and stem cells. Recently, a new set of biosensors was developed to evaluate the specificity of 100 therapeutically relevant GPCRs on hiPSCs (89). Maziarz and colleagues designed a genetically encoded single-molecule BRET biosensor with ER/K linker and YFP (BERKY) to minimize the number and size of sensors while still preserving the native state of the cells being tested. This biosensor permits the detection of endogenous Gα-GTP and free Gβγ without altering GPCRs and their effectors (38).

#### Fluorescence resonance energy transfer

FRET imaging typically uses sensitized emission, fluorescence recovery after photobleaching (FRAP) and fluorescence lifetime imaging microscopy (FLIM). For sensitized emission, the donor fluorophore is illuminated at a specific wavelength and the resulting signal is collected with an emission filter selected for the donor and acceptor fluorophores. However, crosstalk between fluorophores is a common issue, and it is common practice to conduct exhaustive control experiments to determine the presence of FRET (90). Various GPCR sensors have been developed based on this approach, including real-time quantification of cAMP (30, 91), mapping of cAMP around the GPCR (41), measurement of G_α13_ activation in single cells (92), and determination of Rho GTPase activity (93). FRAP is a more accurate method for determining FRET, but it is limited to a single measurement. As a portion of energy from the donor is transferred to the acceptor during FRET, donor fluorescence is extinguished. Photobleaching of the acceptor fluorophore irreversibly eliminates the FRET effect and boosts the donor fluorescence. Mösslein and colleagues utilized FRAP to show a ligand dependent steady-state affinity of β arrestin-3 at a class A GPCR (i.e., μ-opioid receptor) in cells (29).

FLIM is widely regarded as the most comprehensive technique for FRET. It is based on a pulsed interleaved excitation scheme that permits more precise fluorescence lifetime measurements and the identification of regions in the cell containing donor and acceptor fluorophores (94). This approach helps to minimize issues caused by donor emission leakage, photobleaching, and direct excitation of the acceptor, making FLIM the preferred choice over other fluorescence imaging techniques, despite being relatively slow (95). Consequently, FLIM has been utilized in numerous studies to detect more subtle alternations in GPCR structure (47).

A smaller fluorescent probe, fluorescein-arsenic hairpin binder (FlAsH), has been developed as a FlAsh-FRET sensor to reduce the size of the FRET donor-acceptor pair (36, 96). A six-amino acid length peptide containing a tetracysteine motif has been inserted into target proteins; this peptide binds the FlAsH dye specifically, thereby generating a FRET signal between adjacent fluorophore pairs. Compared to the cyan fluorescent protein (CFP)/GFP FRET sensor, the signal amplitude of the CFP/FlAsH FRET sensor is nearly five times higher. Furthermore, dual site-specific and orthogonally labeled FRET sensors, such as FlAsH/ReAsH (resorufin arsenical hairpin binder), can be utilized to examine the dynamic interactions of GPCR with downstream effector molecules (97). Although FRET measurements can present challenges, careful selection of fluorophore pairs can aid in achieving reliable results. To avoid problems like saturation and crosstalk, fluorophores of similar brightness and good spectral separation should be chosen (98). Utilizing a lanthanide donor, such as europium, allows for the time-resolved analysis of protein-ligand interactions (time-resolved FRET, TR-FRET). Typically, the integration delay between the initial donor stimulus and the point of detection for TR-FRET is 60 μs. This delay permits for the background fluorescence to diminish and the recording of the signal specific to the binding event between the labeled proteins. Through TR-FRET, accurate measurements of the binding of the adenosine A_2A_ receptor to various compounds has been validated (68), and the oligomer formation of C-X-C motif chemokine receptor 4 (CXCR4) and atypical chemokine receptor 3 (ACKR3) has been characterized (99). Several commercially available toolkits designed by CisBio to interrogate GPCR signaling also rely on TR-FRET (100–102).

#### Diffusion-enhanced resonance energy transfer

Diffusion-Enhanced Resonance Energy Transfer (DERET) allows monitoring of GPCRs labeled covalently to a cell-impermeable SNAP-Lumi4^®^-Tb as it traverses the cell membrane by measuring the disappearance of donor luminescence following the addition of a free energy acceptor. Conversely, the energy transfer to the acceptor is reduced following constitutive or agonist-induced internalization of the receptor decreasing DERET (103). As DERET signals are particularly suitable for characterizing endocytosis of cell surface receptors, this technique has been widely employed to investigate the effect of ligand-dependent GPCR internalization (42, 103) and β-arrestin recruitment (72).

### Protein complementation assay

Protein complementation assay (PCA) has been used to study the interactions between proteins (104). In these tests, a reporter protein with enzymatic or fluorescent properties is separated into inactive or non-fluorescent fragments, which are then fused to potential interacting proteins. When these fragments are brought together, they spontaneously reassemble into an active enzyme or fluorophore. This technique does not formally demonstrate direct protein-protein interactions, but it does indicate colocalization of the GPCRs of interest because it relies on the “interaction” of the fused partners. Bimolecular Fluorescence/Luminescence Complementation (BiFC/BiLC) and -galactosidase complementation assays are included in PCA and will be subsequently described.

### Bimolecular fluorescence complementarity

The BiFC technique has been refined by incorporating fluorescence proteins. This involves splitting fluorescence proteins at specific sites to generate two non-fluorescent polypeptides (VN and VC), incapable of self-assembly into a fluorescent active site. The fragments are subsequently fused to two proteins that potentially interact. If the two target proteins interact, the incomplete fluorescent protein fragments bind to form an active fluorescent protein that emits fluorescence upon excitation, thereby indicating that the two target proteins have interacted. The BiFC system was initially established on the basis of green fluorescent protein (GFP) residues labeled with antiparallel leucine zippers (105) for proper folding and fluorescence recovery of GFP. Subsequently, enhanced GFP (eGFP) was split into Nterminal-eGFP and C terminal-eGFP fragments to be linked to alpha factor receptors (Ste2p) for homodimerization detection (106). A tripartite GFP system was further proposed to improve GFP folding and self-assembly (107). Apart from GFP, the YFP (108) and EYFP (S65G, S72A, T203Y) variants (109) have been used to improve BiFC. BiFC based on YFP was also utilized in the GPCR dimerization study, including the Neuropeptide Y receptors (110).

BiFC has the benefits of quick results and low background noise. However, it is sensitive to temperature and has issues with self-assembly. Citrine (variant with Q69M mutation) and Venus (variant with F46L mutation) were created to address folding and self-assembly issues, thereby facilitating GPCR dimerization studies at temperature of 37°C (31). Venus is distinguished among the BiFC fluorescence protein splits by its strong fluorescence emission and low background sensitivity, making it the optimal choice for GPCR dimerization detections (111). Despite its improvements, BiFC has unavoidable drawbacks, such as signal lag and the inability to observe the interaction process in real time.

### Bimolecular luminescence complementarity

BiLC is a technique like BiFC, which utilizes split luciferase instead of fluorescent proteins. RLuc, for example, can be split at position 229. NanoLuc Binary Technology (NanoBiT) split NanoLuc into two fragments at position 11, allowing the protein to be labeled with a fragment consisting of only 11 amino acids (112). This diminutive size decreases the probability of spatially interfering protein interactions. The greatest benefit of BiLC is its high signal intensity and signal-to-noise ratio, which enables the detection and quantification of even minute levels of protein interaction without the need to overexpress proteins and potentially disrupt the cellular environment. In addition, the complementary nature of NanoLuc enables it to be used to characterize both binding and dissociation processes. BiLC has been applied in various biological processes of GPCR (9), such as the oligomerization of GPCR (113), recruitment of downstream effectors (114, 115), dissociation of G proteins (114, 115), and activation of G_s_/G_i_ (73). In recent years, NanoBiT has become the technique of choice for numerous studies, demonstrating its applicability and durability.

### Radiological labeling techniques

Radiological receptor labeling is a commonly used technique in receptor binding assays to study the interaction between receptors and their ligands. Developed by Lefkowitz and colleagues in 1970, this technique involves the use of radioactive labeled hormones to measure binding affinity (116). Subsequent studies have employed three radiological ligand binding approaches, including saturation, indirect (competitive, substitution, or modulation), and kinetic binding assays. Radioligands, which are ligands modified to contain radioisotopes such as ^3^H, ^14^C, ^18^F, or ^125^I, are widely used to investigate the binding affinity and kinetic rate constants between ligands and receptors (117). Furthermore, radioligands are often used in conjunction with positron emission topography (PET) imaging to label and study GPCR distribution *in vitro* and *in vivo* (118). Tagged peptides have also been used as radioligands to detect and target their endogenous receptors (119). However, radioligand binding assays require specifically controlled areas and carry health risks. Furthermore, the measurement time for low levels of tritium radiation, for example, can be several hours long (120). Although radioligands can be of great benefit in the study of GPCRs, the lack of radioligands with sufficiently high affinity for some receptors, such as β_3_ ARs, can render them inadequate.

### Genetic labeling techniques

Genetic engineering is utilized extensively for the labeling of intracellular GPCRs and their effector molecules. Small molecules, peptides, and antibodies must all undergo a transmembrane process, which can compromise labeling efficiency. The discovery of fluorescent proteins has made genetic labeling of GPCRs feasible and widespread. Additionally, the precision of genetic engineering allows for the labeling of particular GPCR sites.

#### Over-expression of exogenous proteins

The expression of labeled proteins in living cells is often achieved by producing a fusion protein by joining the labels to the target protein. To accomplish this, the cDNA encoding the desired protein is inserted into a eukaryotic expression vector containing donor or acceptor cDNA. By removing the stop codon between these cDNA sequences, the single fusion protein is expressed in cells (121). This technique has been widely utilized, for example, in ligand binding (64, 74), GPCR oligomerization (122), receptor internalization (123), and signaling studies (124). In addition to the expression of fusion proteins, engineered molecular probes can be transfected into cells to allow measurement of intracellular molecular entities, such as cAMP (125), phosphorylation (126), and other effector molecules (10). However, fusion-based protein tags are limited by the low signal strength, so methods based on genetic engineering to introduce chemical tags have emerged. The most popular of those used is SNAP and CLIP tag technology, designed by the Johnsson and colleagues (127). This technology uses O6-benzylguanine derivatives to covalently bind to the tag and O2-benzylcytosine-based molecules to react irreversibly and rapidly in an orthogonal manner to the SNAP tag (128). These tags have been used in many modifications on GPCRs (129).

**Figure 2:**
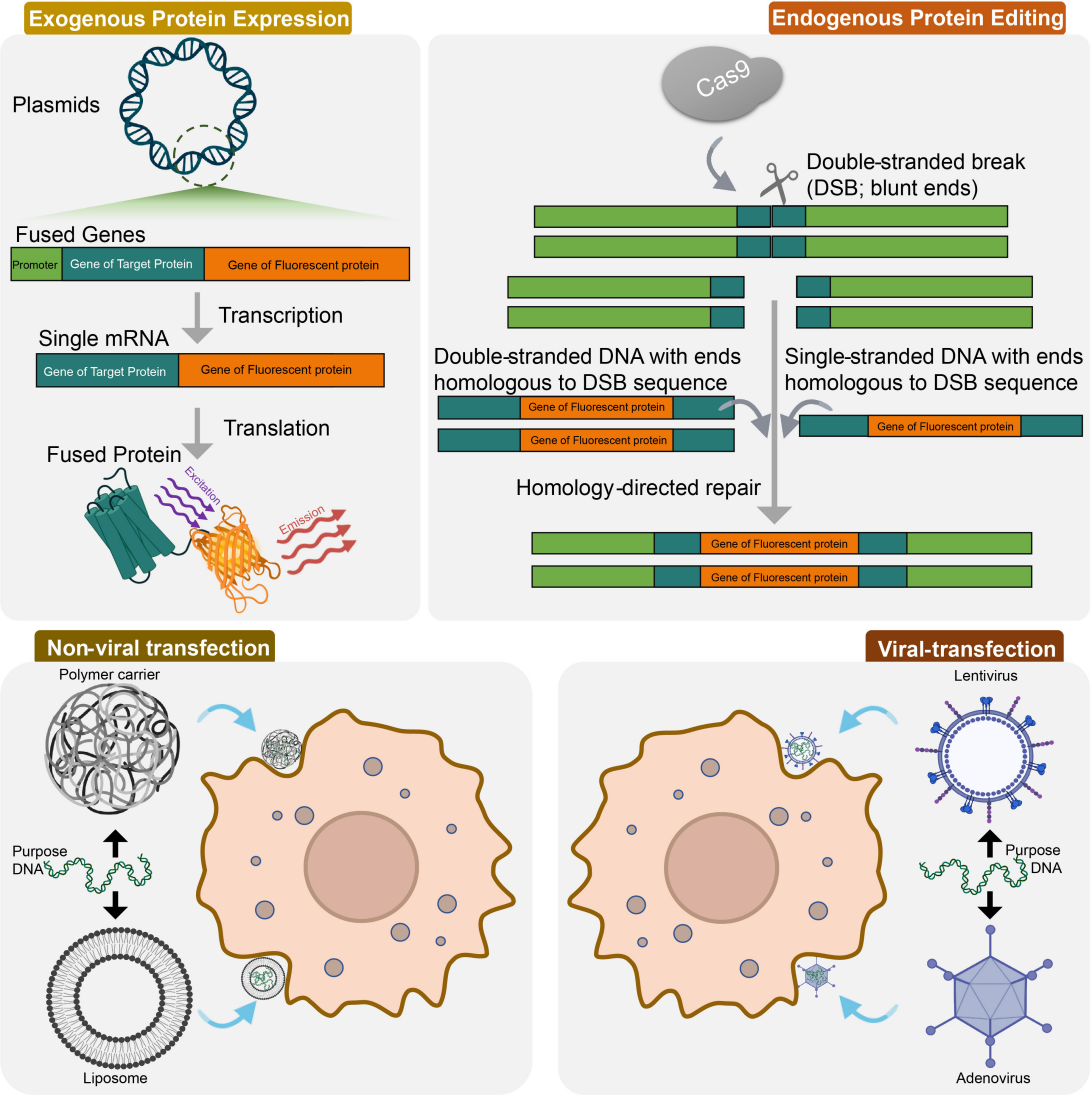
Genetic labeling techniques used in GPCR signaling and spatial distribution. Exogenous protein expression is achieved by producing a fusion protein, which is expressed in cells using a eukaryotic expression vector containing a fluorescent protein gene. CRISPR/Cas9 technology has allowed for the modification of endogenous molecules without the need for exogenous expression. This is achieved *via* homology-directed repair after CRISPR-Cas9-directed double-stranded breaks (DSB). Cell transfection is a commonly used method to express exogenous proteins, and can be divided into two categories: viral vectors and non-viral vectors. Non-viral transfection techniques mainly include polymer carriers and liposomes; viral transfection techniques mainly contains lentivirus and adenovirus.

Cell transfection is a frequently employed technique for expressing exogenous proteins and can be categorized into two groups: viral vectors and non-viral vectors (**Figure 2**). Lentiviral vectors are advantageous because they can infect both dividing and non-dividing cells, have a large gene fragment capacity, and generate long expression times for target genes. For example, lentiviral vectors were used to detect cGMP activity as one of the most important downstream signaling components for various cell types (130). Adeno-associated virus (AAV)-mediated transfection is considered safe and tissue-specific, making it ideal for transfecting primary cells from a wide range of cell types, as exemplified by the successful lacZ reporter gene transfection of primary cardiomyocytes (CMs) using AAV5 (131). AAVs are derived from non-pathogenic defective human microviruses and have a high safety profile, allowing for use at high titers. Currently, there are over 10 distinct serotypes of AAV available, each exhibiting different levels of infection efficiency in different tissues and cells (132).

Non-viral transfection techniques, on the other hand, utilize cationic liposomes and cationic polymers. Cationic liposomes consist of lipids with a cationic head group and one or two hydrocarbon chains, with the cationic head group forming an electrostatic complex with negatively charged nucleic acids. These complexes can subsequently enter cells through endocytosis. Cationic polymers demonstrate a wide host range, ease of handling and low cytotoxicity, making them a desirable choice for use. They form complexes between a positively charged polymer and a negatively charged nucleic acid phosphate group, which bind to a negatively charged polysaccharide on the cell surface and enter the cell by endocytosis. Since 1995, Polyethylenimine (PEI), an organic macromolecule with a strong cationic charge, has been utilized for DNA transfection (133). It has been used for intracellular GPCR labeling, such as for the histamine RLuc8-modified H1 receptor and Venus-modified GPCR kinase (GRK) constructed in HEK293T cells (134)). To address the limitations of multiple plasmid cotransfection, G-CASE (135) was developed, which encodes Gα, Gβ, and Gγ on a single plasmid. This technique links the cDNAs of Gβ and Gγ to the amino acid sequence of the viral T2A peptide by subunit attachment, and the co-expression of Gα is promoted by an upstream internal ribosome entry site (IRES). This eliminates the differences in external expression levels between G proteins and increases the reliability of the assay by obtaining more physiologically relevant receptor/expressions. The choice of transfection method depends on the target cell type and desired application.

#### Editing endogenous proteins

Overexpression of GPCRs and their effector molecules hinders the ability to distinguish patient-specific drug responses based on hiPSCs, as differences between cell types and origins are lost. The introduction of CRISPR/Cas9 technology has enabled the modification of endogenous molecules in cell types carrying specific genetic information, such as primary cells and hiPSCs, without the need for exogenous proteins. CRISPR/Cas9 has allowed for the successful insertion or deletion of genes *via* non-homologous end joining (NHEJ) and homology-directed repair (HDR) following CRISPR-Cas9-directed double-stranded breaks (DSB) (136) (**Figure 2**). For example, protein-Nluc fusions have been produced under endogenous promotion *via* HDR, which makes it possible to observe the interaction between the genome-edited protein and an externally expressed protein *via* BRET. Additionally, CRISPR/Cas9 has been used to label the C-terminus of β-arrestin2 with a modified 11-amino acid NLuc fragment in HEK293 cells (69), as well as to label the N-terminus of the ACKR3 in HeLa cells (137, 138). This type of endogenous modification is essential for hiPSC-based studies as it preserves the natural expression level of the proteins (139). This method permits the study of GPCRs in their native cellular context, with expression levels that more accurately reflect endogenous levels. Additionally, this method preserves the relative abundance of endogenously expressed interacting and dimerization partners (140), which can provide valuable insights into receptor organization, ligand binding, and signal transduction. However, from studies based on embryonic stem cells (ESCs), it can be determined that the efficiency of HDR-based knock-in in human ESCs is about 0.06-0.36%. Knock-in by NHEJ-based strategies, although showing higher efficiencies of about 0.83-1.70% in human ESCs (141), still struggles to meet the requirements of GPCR imaging studies. Additionally, although there is evidence of the potential of the microhomology-mediated end joining (MMEJ) repair pathway in mediating targeted knock-in of large DNA (142), there are currently no reports of MMEJ-based labeling in human ESC/iPSCs.

### Chemical labeling techniques

#### Ligand-directed labeling

**Figure 3:**
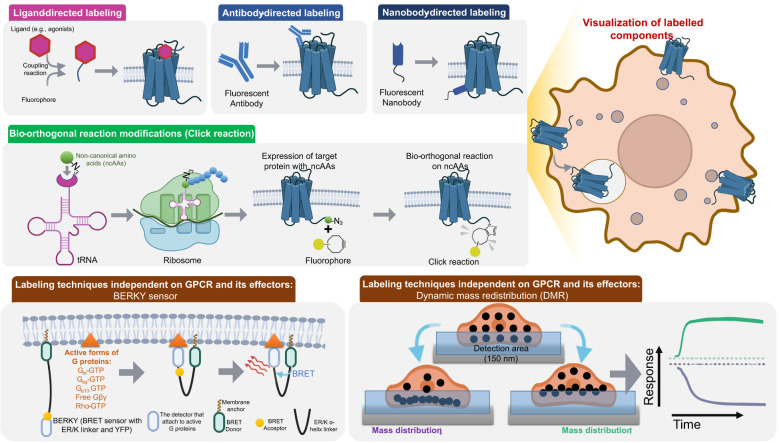
Chemical Labeling Techniques used in GPCR signaling and spatial distribution. This includes Ligand-directed labeling, Antibody-directed labeling, Nanobody-directed labeling and Bio-orthogonal reaction modifications (Click reaction). Additionally, two innovative label-free detection methods, BERKY sensor and DMR, are also presented. Ligand-directed labeling, Antibody-directed labeling and Nanobody-directed labeling are realized through fluorescent molecules with high affinity to the target protein. Bio-orthogonal reaction modifications modifies the target protein with Fluorophore through the introduction of ncAAs using a specific tRNA and codon, followed by Click reaction. BERKY is composed of an ER/K helix linker and BRET proteins and is capable of specifically and sensitively detecting endogenous active forms of G proteins. DMR is based on the principle that when proteins interact, their mass will move together due to weak bonds such as hydrogen bonds and electrostatic forces.

Fluorescent GPCR ligands are a novel alternative to radioactive GPCR ligands that provide a safer and more diverse selection for molecular pharmacology and drug discovery applications, such as the localization of receptor distributions, live cell imaging, and real-time analysis of ligand-receptor interactions (**Figure 3**). Fluorescent probes are typically synthesized by conjugating a fluorophore moiety with a ligand targeting a specific GPCR, sometimes linked by an alkyl chain or an amino acid linker (143). This dual-function molecule permits the non-invasive and selective investigation of the functions and localization of local GPCRs. Even though the concept of fluorescent GPCR ligands is simple, their design presents obstacles. The parent ligand must have pharmacological properties, but the addition of a fluorophore may decrease its binding affinity and selectivity. In addition, the selection of the fluorescent moiety is crucial, as it may introduce functional groups that affect the hydrophobicity, solubility, polar surface area, and charge of the ligand, thereby altering its pharmacological properties (33). Examples of fluorescent GPCR ligands include DALDA-Dansyl and morphinan-Cy5 (144, 145). The cyanine-based fluorescent ligands on the membrane surface can be converted to the long-lived reduced form by treatment with sodium borohydride for GPCR internalization studies. Since the highly polar reducing agent cannot pass through the cell membrane, the fluorescent receptor-ligand complexes in the cytoplasm remain fluorescent under TIRF illumination, allowing its use to quantify internalized GPCRs (59). Fluorescently labeled GPCR ligands can also be used in BRET real-time binding studies with GPCRs (7). In addition, the covalent fluorescent modification induced by the ligand can achieve more stable tracking, as seen with ZM241385-sulfo-cyanine5 (68). Although ligand-induced modification is an alternative to genetic engineering modification that is not feasible for hiPSCs, the inherent drawbacks of fluorescent ligands should also be noted. The intensity of the ligand-specific signal from the ligand is not sufficient for high-throughput pharmacological measurements, and it has indeed been shown to suffer from significant photobleaching in fluorescence microscopy experiments (146).

#### Antibody-directed labeling

Immunofluorescence antibody labeling based on GPCR-specific epitope clusters is a convenient method for GPCR surface labeling (**Figure 3**). Initially, this technique was used to accurately localize the GPCRs on the cell surface with high spatial resolution (147), and later, it was demonstrated to generate FRET signals for direct observation of GPCR dimers on the cell surface (148). The energy transfer between fluorescently labeled monospecific antibodies showed the spatial proximity of GPCRs. This technique has its unique advantages, as conformation-specific antibodies interact with the tertiary structure of the receptor, selectively recognizing its specific folding state (149, 150). However, generating antibodies specific to receptor conformation is challenging. Obtaining homologous receptors with stable correlated conformations is difficult and GPCRs are embedded in the lipid bilayer, preventing most antibodies from approaching the receptor. Therefore, developing conformation-sensitive antibodies is relatively easy for B- and C-family GPCRs with large modular N-terminal structures, but not for A-family GPCRs with shorter N-terminal tails. This technique is effective and convenient, but it is limited by the inability of antibodies to penetrate the cell membrane of live cells. Recently, nanobodies, i.e., single-domain antibody fragments designed from camel heavy chain antibodies, have been developed. In addition to high affinity and specificity, nanobodies (15 kDa) are much smaller than antibodies (150 kDa) and their post-translation modifications are simpler, making them more water-soluble, diffusible, thermally stable, and better expressed in heterologous host cells. Fluorescently labeled nanobody monomers have the same advantages in detecting homodimer receptors. Moreover, the convenience of heterologous expression makes it possible for nanobody engineering to be used as a cellular biosensor to detect conformational changes of GPCRs. GFP-labeled Nb80 is a nanobody specific to the activated β_2_AR that can diffuse into the cytoplasm to detect the activation of the receptor in the cytoplasm, a process that is difficult to achieve with other detection methods. Several conformation-sensitive GPCR nanobodies have been developed, such as those for the μ-opioid receptor and M2-muscarinic acetylcholine (Ach) receptor (151, 152). Typically, two conformation-sensitive nanobodies of the κ-opioid receptor, Nb39 and Nb6, recognize the active and inactive states (62); and Nb80 recognizes the active state of β_2_ AR and was used to analyze the effect of vascular endothelial growth factor receptor 2 on β_2_ AR activation (50). The application of nanobodies is beneficial for the detection of GPCRs, as it reduces the modification of GPCRs. Nevertheless, their application in GPCR functional detection is still limited due to the limited number of nanobodies available for GPCRs, as well as the potential impact of intellectual property protection.

#### Bio-orthogonal reaction modifications (click reaction)

The concept of click chemistry (CC), first proposed by Barry Sharpless and Morten Meldal, is a chemical synthesis method for rapidly and efficiently synthesizing useful new molecules based on carbon-heteroatom bonds (C-X-C) (153, 154). By expanding click chemistry to labeling live cells without interfering with native cellular biochemical reactions, Carolyn Bertozzi introduced the field of bioorthogonal chemistry (155) and was awarded the 2022 Nobel Prize in Chemistry (with Barry Sharpless and Morten Meldal). Strategies for bioorthogonal labeling, such as the Diels-Alder reaction between cyclooctynes (Sph) and diazirines (DpTz), have become promising methods for selectively modifying GPCRs (156). This labeling method introduces bioorthogonal reaction sites into the organism by non-canonical amino acids (ncAAs) mutagenesis, allowing for the selective modification of certain chemical functions in GPCRs (157) (**Figure 3**).

Using bioorthogonal chemistry has advantages, as it can minimize interference with GPCR function while allowing for flexible selection of the regions to be modified. For example, a non-natural amino acid incorporation strategy was used to introduce 4-azido-L-phenylalanine at amino acid 548 in mouse metabotropic glutamate (mGlu) receptor 2 and a Cy5 was introduced on mGlu receptor 2 through a copper-catalyzed azide-alkyne click reaction as a fluorescent modification (35). At the same time, the copper-free click chemistry between strained alkene amino acid and fluorescent quinoxaline probes also achieved the fluorescent labeling of the glucagon receptor (GCGR) (34, 158). The bioorthogonal GPCR fluorescent labeling induced by these reactions can be used for FRET experiments to study the interaction between ligands and receptors, as well as to detect GPCR oligomerization, and even to measure the individual distances between ligands and receptors as constraints to simulate the receptor-ligand interaction (159). Langenhan and Schulte utilized non-canonical amino acid labeling in combination with either FRET or BRET donors to monitor extracellular conformational dynamics of adhesion and Frizzled GPCRs, respectively (160). By using these innovative approaches, they were able to gain insights into the conformational changes that occur during receptor activation and signaling. These studies provide valuable information on the molecular mechanisms underlying GPCR function and have potential implications for drug discovery. These developments emphasize the potential of bioorthogonal modification to further understand GPCR function.

### Labeling techniques independent on GPCR and its effectors

The development of label-free technologies, or technologies independent of the modifications on GPCRs and their downstream effectors, is of great significance. As the existing technologies rely on modification of GPCRs or downstream effectors for monitoring the dynamics of effector recruitment and activation, inevitably yielding data inconsistent with the native states of cells, researchers have been striving to obtain effector response signals directly without modification of the monitored target.

#### The BRET sensor with ER/K linker and YFP

Recently, Maziarz and colleagues developed a novel biosensor, BERKY, independent on labeling of GPCR and its effectors (38) (**Figure 3**). This biosensor is composed of an analyte-targeted ER/K helix linker and a BRET pair, Nluc and YFP. The responsive conformable structure of the ER/K helix allows proximity between the two proteins at both ends, facilitating energy transfer from Nluc to YFP, leading to a measurable increase in YFP fluorescence. BERKY is capable of specifically and sensitively detecting endogenous Gα-GTP in living cells, free Gβγ, and Rho-GTP.

The primary advantages of this single-molecule BERKY biosensor are that it can detect the activity of trimeric G protein in living cells without the need for labeling or overexpressing a modified GPCR. This could aid in better understanding the function of G protein in its native state, the interaction between G proteins and GPCRs, and the development of clinically relevant GPCR ligand drugs based on clinically relevant samples. In addition, the BERKY probe enables a more sensitive and precise tracking of endogenous G protein activity and its temporal variation in real time.

#### Label-free dynamic mass redistribution assay

Label-free dynamic mass redistribution (DMR) assay is an innovative method for studying dynamic interactions between proteins without the need for any modifications to intracellular components (**Figure 3**). This technique is based on the principle that when proteins interact, the mass of both proteins will move together due to the force of weak bonds, such as hydrogen bonds and electrostatic forces, that enable the proteins to move in unison without direct contact. Guided wave grating resonance is a popular technique for DMR in the vicinity of a sensor surface. This technique involves the measurement of a shift in the reflected wavelength, which is directly related to the magnitude and direction of the DMR. Impedance-based sensing is another approach that can be used to detect DMR, and it offers certain advantages over waveguide-based sensing, such as ease of use and lower cost. In guided wave grating resonance, the shift in the reflected wavelength is characterized as p-DMR or n-DMR depending on whether the mass is moving towards or away from the sensor surface, respectively (161).

This method can easily be adapted to different cellular backgrounds (adherent or suspension), including primary human cells. Real-time recordings can be conducted in a 384-well microtiter plate and completed within two hours or, alternatively, they can be extended to several hours (162).

The DMR technique does not require any additional labeling of the proteins, as ultrasound pulses are used to mix and interact with the proteins in the solution. This makes it possible to measure the degree of interaction between the two proteins and any changes in their conformation without the need for any additional labeling. Recent research in the GPCR field has highlighted the potential of the DMR technique in understanding receptor biology and compound behavior. For instance, by using an Epic^®^ reader for high throughput screening of antagonists (163), the effects of various sweet taste receptor agonists and other modulators were recorded by measuring changes in DMR. This showcases the versatility of the DMR technique, as it can be employed to study a broad array of protein interactions. Moreover, DMR offers a direct measure of Gαi_/o_-coupled GPCR activation without the need to pharmacologically manipulate the adenylyl cyclase–cAMP module to probe for Gαi_/o_ activity (42, 162), and deconvolute GPCR signaling in live cells (164).

## Methods for GPCR signaling detection

The methods used to monitor GPCR signaling and to describe reacting components of the signaling pathway can be divided in three classes: Techniques that monitor one single component of the pathway, techniques utilized for describing the interaction of two distinct components of the signaling pathway, and methods that include more than two different components involved in mediating the receptor signal.

### Single component detection methods

#### Cyclic adenosine monophosphate

After GPCR stimulation, cAMP is a pivotal second messenger and a crucial marker of cellular responses. Several commercial cAMP detection kits, including MSDTM, HTRF^®^, and AlphaScreen, have been developed. These kits facilitate binding reactions in which endogenous cAMP and a cAMP tracer compete for a binding site for detection. For FRET (165) or BRET (5, 166) based assays, an genetically encoded fluorescent cAMP sensor with a cAMP binding domain flanked by two different fluorophores (i.e., Epac1-camps) has been utilized. To examine the spatiotemporal relationship between GPCRs and cAMP in living cells, a cAMP receptor based on Epac1 was recently developed (41, 167), and being further developed to achieve better signal to noise ratio by Jalink and colleagues (168). By introducing a 30-nm single-alpha-helical domain linker based on ER/K repeats between glucagon-like peptide-1 receptor (GLP-1R) and Epac1-camps, a “nano ruler” was created to measure the cAMP-containing domain surrounding the receptor. Upon binding of glucagon-like peptide-1 (GLP-1), the GLP1-receptor-related cAMP domain was measured to have a radius of approximately 60 nm. Using such sophisticated distance sensors provides insight into the size of the cAMP domain surrounding an activated GPCR, which aids in the comprehension of the intricate spatial regulation of receptor signaling.

#### Intramolecular conformational changes in GPCRs

**Figure 4:**
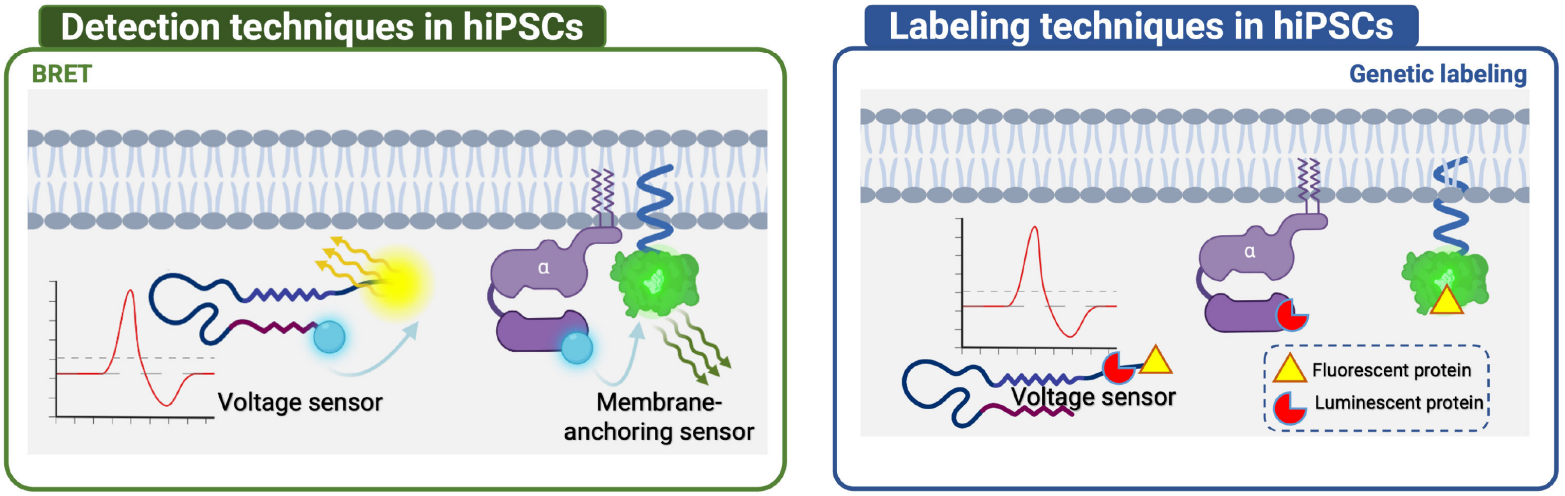
Detection and labeling techniques in hiPSCs. In hiPSCs, limited techniques were applied, including BRET for Voltage sensor and Membrane-anchoring sensor detection, and Genetic labeling for Voltage sensor, Membrane-anchoring sensor, Gα labeling.

Intramolecular GPCR conformation sensors consist of a GPCR and two fluorescent tags. These sensors can reflect the active state of GPCR induced by agonists, including agonist-induced conformation changes, and the movement between sixth transmembrane helix and adjacent intracellular regions during agonist-induced activation (43). Furthermore, the dynamic properties of these sensors are fast and compatible with the rapid physiological responses triggered by GPCRs; activation time constants of α_2_-AR and parathyroid hormone (PTH) receptor sensors were τ < 40 ms and 1 s, respectively (169). Subsequently, many similar GPCR activation sensors have been reported, including β_1_ and β_2_ ARs (44), adenosine A_2A_ receptors ARs (170), M_1_, M_2_, M_3_, and M_5_ muscarinic receptors (45, 171). Many GPCRs have been observed to exhibit normal characteristics when two large fluorescent proteins are linked to the receptor. The attachment of a fluorescent protein to the third intracellular loop of GPCRs has been reported to disrupt G protein coupling in some cases. For example, inserting CFP and YFP into human adenosine A_2_ receptor eliminates the coupling with adenosine cyclase. While a recent study using a coupling-capable M3R conformational biosensor demonstrated an exception to this statement (172). Using the CFP/FlAsH-tetracysteine system is an alternative to fluorescent protein, which gave a five times larger FRET signal induced by the agonist and completely normal downstream signals (96). Similar results were also obtained for the mouse α_2_ ARs. This suggests that the small-sized FlAsH-tetracysteine labeling (~1 kDa) may have a decisive advantage in monitoring structural changes within GPCR molecules (96). Another GPCR activation-based (GRAB) sensors were developed by inserting circularly permuted green fluorescent protein (cpGFP) into the third intracellular loop of a GPCR. Upon agonist binding, the third intracellular loop undergoes a conformational change that changes the fluorophore environment of cpGFP, resulting in a fluorescence change (**Figure 4**). These sensors have been developed to detect dopamine (173), endogenous opioid peptides (173), serotonin (174), noradrenaline (173), and Ach (175).

#### Intramolecular conformational changes in effectors

The GPCR effector, β-arrestin, has been extensively studied in terms of its conformational changes. The discovery of the proximity between the N- and C-termini of β-arrestin was achieved through a BRET-based biosensor (176). Subsequent studies have increased the brightness and broader spectral separation by using Nluc and cyan-emitting fluorescent protein (CyOFP1) (177)

Furthermore, the introduction of the R170E mutation allows the sensor to detect various stages of activity of β-arrestin2. This enables monitoring of conformational changes of β-arrestin at different stages of GPCR activation (177). Molecular Flash-BRET sensors using RLuc and Flash pairs or Flash-FRET sensors using CFP and Flash pairs have also been developed. These have been used to demonstrate different conformational changes of β-arrestin induced by different ligands and GPCRs (178, 179). In summary, these studies provide important insights into the role of β-arrestin in GPCR activation and its conformational changes.

### Two components

#### Effector binding assays

G proteins are heterotrimers composed of α, β, and γ subunits that form a heterotrimeric structure anchored by lipids in the plasma membrane. Upon binding to its cognate GPCR, the ligand induces a conformational change releasing its specific G protein, which subsequently triggers second messengers activating downstream intracellular events. Fluorescent biosensors for the usage in RET or PCA based assays have been developed to particularly study the binding and dissociation of G proteins at its receptor. Gαs and Gα13 activation have been measured by FRET between the yellow fluorescent protein labeled Gα and the Gβγ subunit labeled with cyan fluorescent protein (46, 92). FRET between Gαq-eCFP or eCFP-Gβγ and type 2 bradykinin receptor (B2R)-eYFP has been used to monitor the binding and dissociation of the stable B2R-Gαq-Gβγ ternary complex in quiescent cells (180). To quantify receptor internalization, a BRET sensor has been used to detect the recruitment of β-arrestin to active GPCRs in living cells by using RLuc and GFP as BRET donors and acceptors, respectively (181). FRET sensors have been used to measure the recruitment kinetics of β-arrestin, such as CFP-tagged parathyroid hormone 1 receptor (PTH1R) and YFP-tagged β-arrestin2 to detect the time delay of β-arrestin2 recruitment to PTHR after receptor activation (182). Compared to BRET sensors, these methods are more suitable for visualizing the dynamics of GPCRs and β-arrestin in cells with high temporal resolution. Additionally, to avoid the large molecular weight of modified G proteins, mini-G proteins composed of only the sequences required for GPCR coupling have been designed and fused with fluorescent proteins to reduce the molecular weight and avoid the occlusion effect (54).

#### GPCR dimerization

Still controversial but suggested by a number of authors, GPCRs may form dimers, with C-family GPCRs being shown to exclusively act *via* dimers (183). Homodimers of C-family GPCR (e.g., mGlu receptor), as well as heterodimers (e.g., gamma-aminobutyric acid (GABA) type B receptor subunit 1 and 2), have been detected in experiments using FRET and BRET techniques (184). FRET assays have been used to detect the formation of heterodimers of A-family GPCRs, such as the oxytocin receptor (OTR) and the prostaglandin E2 receptor, (EP2) (4), which produces a flip effect on subsequent signal transduction. Intra- and intermolecular FRET sensors have been utilized for mGlu receptors, in conjunction with photo-uncaging, to investigate the stepwise activation mechanisms in class C GPCRs (185).Similarly, BRET has been used to detect dimerization of the A-family histamine H3 receptor (H3R) (28). It is important to note, however, that the decrease in distance between GPCRs may be due to receptor interference, rather than heterodimerization. Furthermore, overexpression of receptors may artificially produce GPCR dimers (186), making it necessary to use caution when confirming GPCR dimers by imaging. Usually, the amount of G protein binding or the crystal structure of the dimer needs to be further verified.

### Multiple components

The light-activation and light-transformation properties of mIrisFP fluorescent protein have been employed to construct a mIrisFP BiFC and a three-fragment fluorescent complementation system (TFFC). By combining BiFC and TFFC with light-activated localization microscopy, the super-resolution imaging of protein-protein and protein-tetramer subunit interactions with spatial resolution has been achieved down to 40 nm (40). This enabled researchers to observe the different distribution patterns of heterotrimers of G αβγ in subdiffractional-limited cellular space, as well as the dynamic process of dissociation of the αs subunit and βγ heterotrimer. Moreover, the combination of BiFC and BREThas also been applied to illustrate the interaction between multiple components such as higher-order GPCR oligomers (187).

### Activated effector assays

The on/off state of G proteins is determined by their nucleotide-binding status, with Guanosine diphosphate (GDP) bound G proteins in the off state and Guanosine triphosphate (GTP) bound G proteins in the on state. This switch is mainly regulated by nucleotide exchange and GTPase, which is catalyzed by GPCR. The subsequent release of the free Gβγ dimer is usually monitored, as it avoids modifying the GPCR or Gα subunit. The detection of free Gβγ was first studied using FRET and BRET techniques by Hollins and colleagues (188). It was found that fusion proteins containing the c-terminus of GPCR kinase 3 (GRK3ct) (fused with cerulean or RLuc) could bind to Venus-tagged Gβγ dimers, leading to changes in FRET or BRET. This has since been applied in multiple studies to measure intracellular Gβγ activation in response to different stimuli (189). The pioneering work of the Bouvier and Garcia-Marcos contributed to the development of activated effector assays which utilize the detection of active G protein subunits (BERKY from Garcia-Marcos (38) and the translocated downstream proteins (such as PKN-RBD and p63RhoGEF) introduced by the Bouvier and colleagues) (190).

A new TGFα shedding assay represents a significant advance in the GPCR field because it provides an accurate and versatile method for detecting GPCR activation, particularly Gα_12/13_-coupled signaling poorly characterized by conventional assays. The assay measures ectodomain shedding of a membrane-bound preform of alkaline phosphatase-tagged TGFα (AP-TGFα) and its release into conditioned medium (191). The AP-TGFα shedding response occurred almost exclusively downstream of Gα_12/13_ and Gα_q_ signaling, and the assay can detect 104 out of 116 human GPCRs.

This new assay is particularly useful for identifying ligands for orphan GPCRs and determining the mode of ligand-GPCR interactions. The ability to identify previously uncharacterized GPCRs and study their signaling pathways will aid in the development of new drugs and therapies. Additionally, the TGFα shedding assay provides a single-format method to detect multiple GPCR signaling, which will streamline research and increase efficiency. One limitation of the TGFα shedding assay is its limited throughput and sensitivity. However, the assay’s versatility and accuracy more than compensate for this limitation, making it a valuable tool for researchers in the field of GPCR research.

### Transcriptional readouts

Transcriptional readouts constitute another valuable tool for studying GPCR signaling. These assays utilize reporter genes (i.e., luciferase activity) controlled by specific promoter elements activated through classical GPCR signaling molecules, including cAMP response element (CRE), nuclear factor of activated T-cells response element (NFAT-RE), serum response element (SRE) and serum response factor response element (SRF-RE). One such assay utilizes CRE (cAMP production), SRE (ERK/MAPK activity), NFAT-RE (intracellular Ca^2+^ mobilization), or SRF-RE (RhoA activity) response elements to regulate luciferase expression and monitoring its enzymatic activities with luminescent read-outs (192). These reporter gene assays have simplified the characterization of GPCR/G protein coupling, as they allow decipher G protein activation profiles for various receptors, including exogenous m3 muscarinic receptor and endogenous β_2-_AR receptors in HEK293 cells. Possible applications include potency rankings of agonists and antagonists, high-throughput screening, and the study of receptors with unknown coupling mechanisms. These assays provided new, important insights into the mechanisms of adhesion GPCR signaling and but have also advanced to measure adhesion GPCR autoproteolysis (193).

In summary, reporter gene assays have revolutionized the study of GPCR signaling pathways and constitute a valuable tool for drug discovery efforts and have numerous applications in the field of GPCR research.

## Cell-based assays

**Figure 5:**
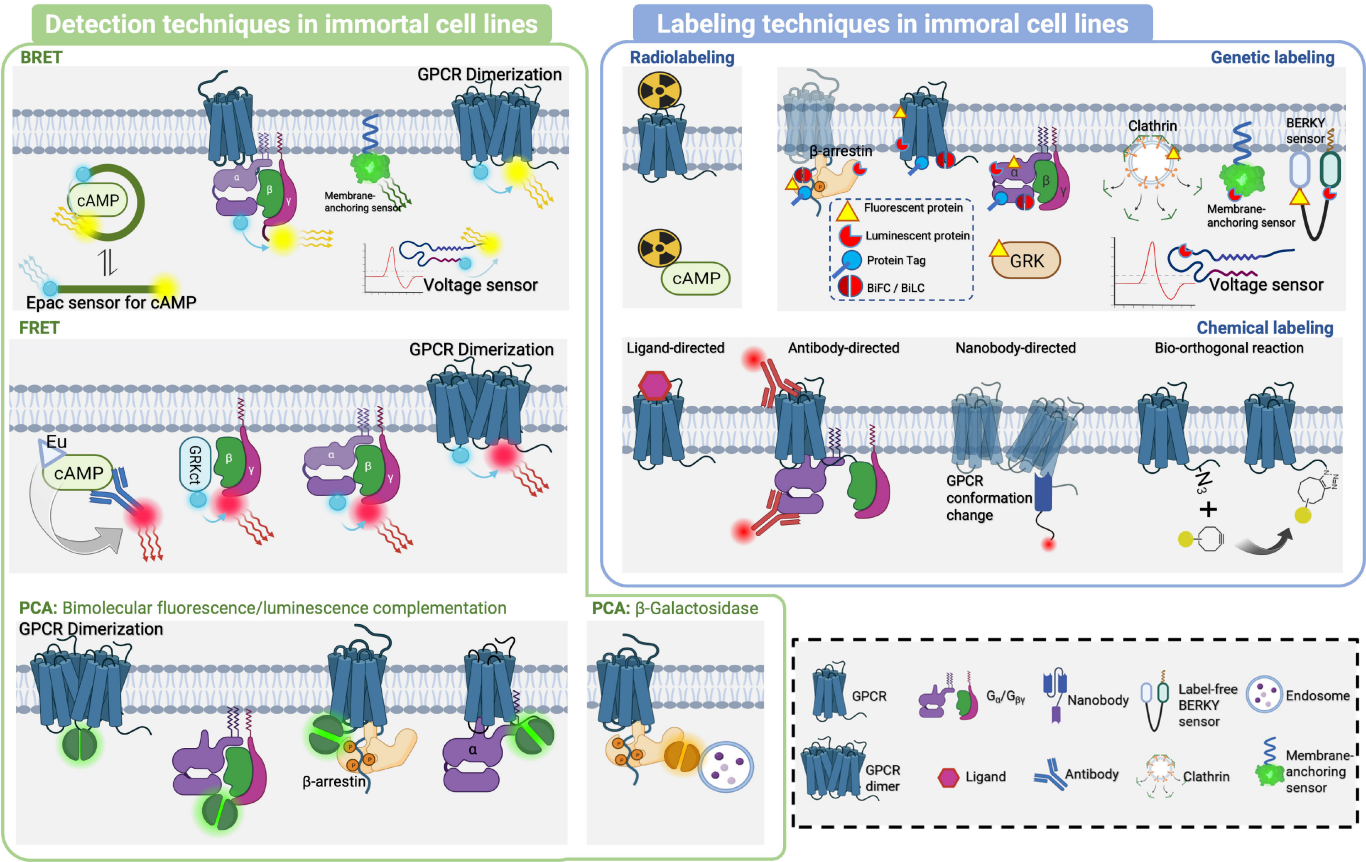
Detection and labeling techniques in immortal cell lines. In the immortal cell lines, BRET, FRET and PCA were used for detection of GPCR-related protein interactions. In BRET, classical ones included Gα/Gβγ or GPCR dimer binding/dissociation to generate BRET signal change. Membrane-anchoring sensor was used to detect effector molecules recruited to the cell membrane, as well as peptide sensors of conformational change, including Epac sensor to recognize cAMP, and Voltage sensor to sense voltage-sensitive conformational change to generate BRET signal change. The application of FRET has primarily focused on detecting binding or dissociation events involving various protein complexes such as GRKct/Gβγ, Gα/Gβγ, and GPCR dimers. In PCA, Bimolecular fluorescence/luminescence complementation was used to detect interactions of GPCR dimer, Gα and Gβγ, GPCR and β-arrestin, GPCR and Gα. β-Galactosidase splits was used to detect interaction of endosome and β-arrestin. In immortal cell line, Radiolabeling was used to label GPCR and cAMP, while Genetic labeling was used to label with Fluorescent protein, Luminescent protein, protein tag, Bimolecular fluorescence/luminescence complementation splits on β-arrestin, GPCR, Gα and Gβγ, Clathrin, Membrane-anchoring sensor, BERKY sensor, Voltage sensor. Chemical labeling was mainly achieved by ligand labeling of GPCR, antibody labeling of GPCR and Gα, nanoantibody labeling of GPCR conformational change, and Bio-orthogonal reaction labeling of special sites of GPCR.

This section compares the detection and labeling techniques applied to immortal cell lines and hiPSCs (**Figures 4**, **5**). Immortal cell lines employ several techniques to detect GPCR-related protein interactions, including BRET, FRET, and PCA. BRET is used to detect Gα/Gβγ or GPCR dimer binding/dissociation, as well as effector molecules recruited to the cell membrane and conformational changes in GPCR. FRET is mainly used to detect the binding or dissociation of GRKct Gβγ, Gα and Gβγ, and GPCR dimers. PCA includes bimolecular fluorescence/luminescence complementation and β-galactosidase splits. Labeling techniques such as radiolabeling, genetic labeling, and chemical labeling are also employed in immortal cell lines. In contrast, hiPSCs have limited techniques for detection and labeling, including BRET, genetic labeling, and chemical labeling (**Tables 3**, **4**).

**Table 3:** GPCR detection techniques used in immortalized cell lines and iPSCs.

Technique		Target	Cell line	hiPSC	Purpose	Ref.
BRET	BRET-1	κ-opioid receptor/nanobody	Sf9		Conformational change	(62)
		Histamine H3 receptor dimer (H3R dimer)	HEK293		Dimerization	(28)
	BRET-3	Exchange protein activated by cAMP	HEK293		cAMP measurement	(123)
	ebBRET	G proteins/membrane sensor	HEK293	iPSC-CM	Receptor-effector interaction	(12)
		Angiotensin II type 1 receptor (AngII-1R)/β-arrestin	HEK293		Receptor-effector interaction	(66)
FRET	Normal FRET	G protein-coupled receptor kinase 3 C-terminal (GRK3ct)/Gβγ	HEK293		Kinase-effector interaction	(188)
		Gα_q_/Gβγ	HEK293		G protein interaction	(180)
		5-hydroxytryptamine 2C receptor (5-HT2C)/Golgi	HEK293		Receptor-Golgi colocalization	(53)
		Metabotropic glutamate receptor 2 dimer	CHO		Dimerization	(57)
		Genetically encoded voltage indicators	HEK293	iPSC-CM	Voltage detection	(194, 195)
	TR-FRET	Cyclic adenosine monophosphate	HEK293		cAMP measurement	(38)
PCA	BiFC	Dopamine D_2_ receptor/α_2_ adrenergic receptor	CAD		Dimerization	(111)
		Gβ/Gγ	HEK293; Vero		G protein interaction	(40, 189)
	BiLC	Gα_i_/Gγ	HEK293		G protein interaction	(115)
		Gα_s_/Gγ	HEK293		G protein interaction	(73)
		mini-Gα_s_,-Gα_i_,-Gα_q_/β-arrestin	HEK293		G protein interaction	(72)
	β-Gal	β-arrestin2/ endosomes	HEK293		Effector-endosome interaction	(123)

R-E-Int, Receptor-effector interaction; C-change, conformational changes.

**Table 4:** GPCR labeling, and detection techniques used in immortalized cell lines and iPSCs.

Technique		Target		Cell line	hiPSCs	Purpose	Ref.
Radiolabeling		α_2_ adrenergic receptor		HEK-TSA		Radioligand binding	(85)
		Dopamine D_2_ receptor		CAD cells		Radioligand binding	(111)
Genetical labeling	Fluorescent protein labeling	β_2_ adrenergic receptor		HEK293		Receptor-effector interaction	(46)
		Bradykinin receptor B2		HEK293		Receptor-effector interaction	(29, 179)
		Histamine H3 receptor		HEK293		Localization	(180)
		μ-opioid receptor		HEK293		Dimerization	(113)
		β_2_ adrenergic receptor		HEK293		Location; Dimerization	(28)
		Bradykinin receptor B2		HEK293		R-E-interact	(29)
		Clathrin		HEK293		Receptor-clathrin interaction	(42)
		β-arrestin		HEK293; COS-1		R-E-interact	(20, 29, 43, 50, 67, 182, 196)
		Gα_q_, Gα_s_, Gα_13_		HEK293; Hela		R-E-interact	(38, 46, 180)
		Gβγ		HEK293; Hela		R-E-interact	(38, 180)
		G protein-coupled receptor kinase 3 carboxyl-terminus (GRK3ct)		HEK293		Kinase-effector interaction	(188)
	Overexpression of exogenous proteins	Luminous protein labeling	5-hydroxytryptamine_2C_ receptor	HEK293		Dimerization	(53)
			Gα_s_, Gα_q/11_, Gα_12/13_	HEK293		R-E-interact	(12)
			Gα_i/o_	HEK293	iPSC-CM	R-E-interact	(12)
			β-arrestin	HEK293		R-E-interact	(42)
			Platelet-activating factor receptor	HEK293		Dimerization	(5)
			Vascular endothelial growth factor receptor	HEK293		Dimerization	(50)
			C-terminal tetrapeptide sequence (CAAX)	HEK293	iPSC-CM	R-E-interact	(12)
		Protein tag labeling	β_1_ adrenergic receptor	HEK293		Location	(30)
			β_2_ adrenergic receptor	HEK293		Dimerization	(50)
			5-hydroxytryptamine_2C_ receptor	HEK293		Location	(53)
			β_2_adrenergic receptor	HEK293		R-E-interact	(42)
			α_2_-adrenergic receptor	CHO-K1		Dimerization	(27)
			Gα_i_	CHO-K1		R-E-interact	(27)
			β-arrestin2	CHO-K1		R-E-interact	(59)
		BiFC	Dopamine D_2_ receptor	CAD		Dimerization	(111)
			α_2_ adrenergic receptor	CAD		Dimerization	(111)
		BiLC	Cholecystokinin A receptor	HEK293		Dimerization	(73)
			FK506 binding protein	Hela		R-E-interact	(112)
			Adenylyl cyclase 5	HEK293		Adenylyl Cyclase-effector interaction	(42)
			Dopamine D_2_ receptor	HEK293		R-E-interact	(113)
			mini-Gα_i_	HEK293		R-E-interact	(113)
			Gα_i_	HEK293		R-E-interact	(73)
			Gα_s_	HEK293		R-E-interact	(42)
			β-arrestin	HEK293		R-E-interact	(50)
		Techniques independent on GPCR and its effectors	Bioluminescence resonance energy transfer sensor with ER/K linker and yellow fluorescent protein (BERKY) sensor	HEK293; Hela		R-E-interact	(38)
	Editing endogenous proteins	Fluorescent protein	Genetically encoded voltage indicators	HEK293	iPSC-CM	Voltage detection	(194, 195)
		Luminous protein	β-arrestin	HEK293		R-E-interact	(69)
			C-X-C chemokine receptor type 4	HEK293		R-E-interact	(69)
			Atypical C-X-C chemokine receptor 3	HEK293		C-change	(137)
Chemical labeling	Ligand-directed	Dopamine D_2_ short and long receptors (D_2_SR and D_2_LR)		CHO-K1		Localization	(59)
		μ-opioid receptor		CHO-FlpIn		Localization	(144)
		α_1_A adrenergic receptor		CHO-A1		Localization	(33)
		α_2_A adrenergic receptor		HEK293		Localization	(68)
	Antibody-directed	Ga_q_		HEK293		R-E-interact	(180)
	Nanobody-directed	μ-opioid receptor		Sf9		C-change	(151)
		Muscarinic acetylcholine M_2_ receptor		Sf9		C-change	(152)
	Bio-orthogonal	Glutamate metabotropic receptor 2		HEK293		C-change	(35)
		Glucagon receptor		HEK293		Localization	(34, 158)

R-E-Int, Receptor-effector interaction; C-change, conformational changes.

### Immortal cell lines

Two types of cells are commonly used in eukaryotic protein expression systems: HEK293 cells and CHO cells. Both cell types possess accurate post-translational modification, efficient gene amplification and expression, and high tolerance to shear and osmotic stress. CHO cells are advantageous in that they rarely secrete endogenous proteins, making it easier to isolate and purify target proteins. HEK293 cells, on the other hand, have the benefits of high growth density, fast growth rate, ease of culture and transfection. Consequently, these two cells are widely used in GPCR research due to their excellent performance in eukaryotic protein expression systems.

#### Human embryonic kidney cells

The HEK293 cell line was initially established by introducing sheared adenovirus 5 DNA to primary human embryonic kidney cells *via* transfection and has since been demonstrated to stably express the adenoviral E1A and E1B-55k proteins due to the incorporation of a 4 kbp adenoviral DNA fragment in the chromosome (197). HEK293 cells are particularly attractive for GPCR research as they naturally express 75 different GPCRs together with many G protein signaling regulators (i.e., β-arrestin1 and 2, and GRK3-5). In addition, HEK293 cells are easy to transfect by either viral (130) or non-viral methods (198) and possess an almost complete set of G protein subunits constituting an attractive environment for overexpressing additional GPCRs of interest (199). HEK293 cells have been utilized to answer a multitude of different questions in the past: For example, the Gα_q/11_-knockout HEK293 cells were used to validate dimerization and signal crosstalk of EP2 and OTR (4) while the contributions of endogenous Gα_q/11_, Gαs, Gα12/13, and Gβγ proteins to histamine receptor signaling has been studied utilizing unlabeled DMR detection technique (200). Surface fluorescence of FLAG-β_1_AR or FLAG-β_2_AR expressing HEK293 cells were used to measure receptor internalization (196).

#### Chinese hamster ovary cells

A second immortal cell line widely used in GPCR research are CHO cells, which are characterized by their ability to grow in suspension cultures, their usage in antibody generation, a low rate of contamination with human viruses, the stable integrability of exogenous genes, and finally their excellent suitability for the isolation and purification of target recombinant proteins. CHO cells have been used in a variety of GPCR studies. Due to CHO cells having an exo-endo αIIbβ_3_ signaling response characteristic of platelets, CHO cells are used to assess the proximity between cellular sarcoma tyrosine kinase and integrin alpha-IIb/beta-3 (αIIbβ_3_) (70). Direct local interactions between c-Src and αIIbβ_3_ were reported by BRET and BiFC patterns. Other studies have used CHO cells to study the interactions between apelin receptors and other receptors by BRET and BiFC (201). CHO cells have also been used to determine the difference in agonist-induced internalization ability between the short (D_2_S) and long (D_2_L) isoforms of the dopamine D_2_ receptor (59).

### Human induced pluripotent stem cell-derived somatic cells

#### Generation of h-iPSCs

**Figure 6:**
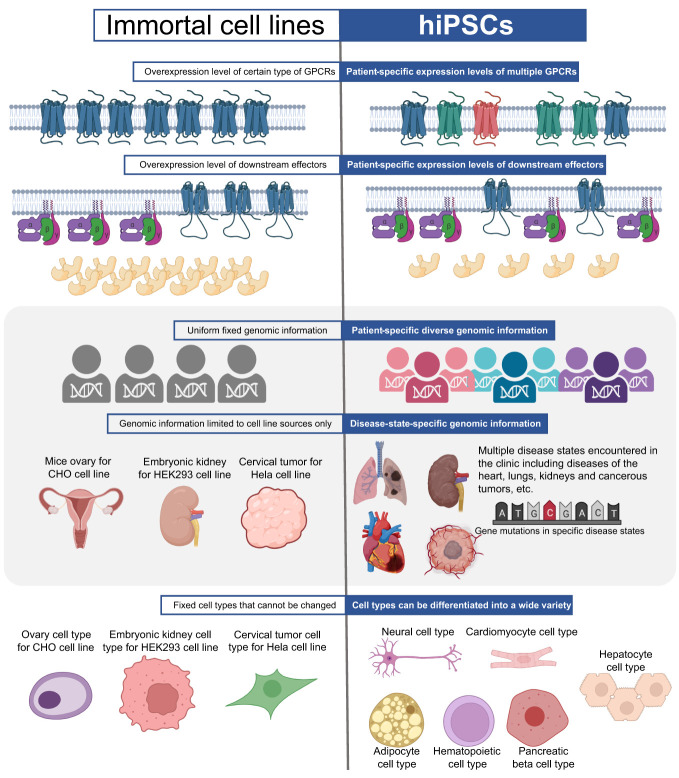
Comparison of hiPSCs and immortal cell lines in GPCR research. Immortal cell lines typically rely on exogenous overexpression of the target GPCR and downstream effectors, whereas the types and expression levels of GPCRs and downstream effectors in hiPSCs are patient-specific and endogenous. Immortal cell lines carry the genomic information of the cell donor, whereas hiPSCs carry genomic information from a variety of patients, providing diverse genetic profiles and representing diseases of the heart, lungs, kidneys and cancerous tumors. Furthermore, the limited cell types available in immortal cell lines restrict the study of heterogeneous responses in different tissue types and cell types, with the common immortal cell lines representing only ovary cell type for CHO cell line, embryonic kidney cell type for HEK293 cell line and cervical tumor cell type for Hela cell line. In contrast, hiPSCs can be differentiated into a range of different cell types, including neurons, CMs, adipocytes, hematopoietic, pancreatic, and hepatic cell types.

The generation of hiPSC from somatic cells has opened new avenues for biomedical research and personalized medicine. HiPSCs can be derived from various sources of somatic cells, such as skin fibroblasts or peripheral blood mononuclear cells (PBMCs), by different methods of reprogramming, such as viral, non-viral, or chemical induction (202). Briefly, the procedure includes isolation and expansion of human fibroblasts and PBMCs, electroporation and generation of hiPSCs, selection and amplification of HiPSCs. hiPSCs can then be differentiated into various cell types of interest, such as neural cells, CMs, adipocytes, hematopoietic cells, pancreatic beta cells, hepatocytes (**Figure 6**).

#### Difficulties in transfection

Transfecting hiPSCs presents a significant challenge, including selecting compatible methods, designing appropriate plasmids, controlling gene expression levels, and avoiding epigenetic changes that could compromise differentiation potential and gene expression. While the transfection efficiency of hiPSCs is often low (203, 204) several groups introduced specific methods to overcome this limitation: Chatterjee and colleagues utilized the GeneJuice transfection reagent for introducing an eGFP containing plasmid into iPSCs resulting in robust and reproducible efficiency without affecting iPSC pluripotency (205). Rapti and colleagues compared the transduction efficiency of recombinant AAV, adenovirus, and lentiviral vectors between both undifferentiated and differentiated hESC and hiPSC lines. The authors found AAV being effective in both cell types, while lentiviral vectors were more suitable for undifferentiated stem cells. Notably, the transduction efficiency of different viral vectors corresponded to the abundance of their respective receptors (206). While genetic modification of hiPSC provides advantages regarding limitless cellular availability, it also requires careful selection and optimization of an appropriate transfection method demands to ensure compatibility with hiPSC to avoid genetic instability or cellular damage. Plasmid design should be carefully planned to ensure pluripotent properties of modified hiPSCs. Generating hiPSCs entails addressing chromosomal abnormalities, genetic instability, copy number variants, and loss of heterozygosity by considering the cellular source, reprogramming method, and culture conditions to reduce the risk of mutation and tumor formation. The safety of reprogramming strategies, as well as genomic and karyotype integrity, must be meticulously evaluated in hiPSC based experiments. Therefore, the effective modification of hiPSC should be carefully considered and weight against methods targeting somatic cells derived from hiPSCs. Careful consideration of these factors is necessary for the effective and safe utilization of hiPSCs in GPCR research.

#### Application of DPCR signaling and spatial distribution techniques in hiPSCs

The application of hiPSCs opened the door for understanding patient specific drug responses and developing more specific therapeutics (**Figure 6**). The need to overexpress engineered G proteins or GPCRs of interest in immortalized cell lines limits the reliability of studies relying on immortalized cell lines, as this may lead to an inconsistent expression ratio of the receptor/effector compared to the native state, resulting in erroneous results (135, 186). Using hiPSCs allows for protein-protein interactions and dynamic studies to be as close to the natural cell environment as possible, especially when using fusion proteins expressed at near physiological levels. In addition, hiPSCs possess a major advantage over immortal cell lines in their capacity to differentiate into various somatic cell types, such as CMs and neurons, which are difficult to access in patients (135, 140). This allows assessing GPCR signaling heterogeneity between various cell types. A proof-of-concept platform based on hiPSCs has been established to facilitate high-throughput drug screening of different induced neuronal cell (iNC) populations (207). This platform utilizes excitatory and inhibitory hiNCs, which are placed in two independent chambers of each well connected by microchannels and can be used to test personalized therapeutic drugs in a cell environment carrying a patient’s genetic background. However, existing studies have not fully utilized the advantages of hiPSCs, and studies using hiPSCs are limited to encoding voltage indicators to characterize membrane potential changes, ACh changes, or visualizing Ca^2+^ changes in the sarcolemma. For example, Bourque and colleagues used AAV6 to transduce the red-shifted RGECO-TnT biosensor in hiPSC-derived CMs, and observed the sarcomere localization fluorescence of RGECO-TnT, and measured several Ca^2+^ transient characteristics, including Ca^2+^ transient amplitude, area under the curve, transient frequency, and transient duration (208). There are also studies using transcription activator-like effector nucleases technology to establish gene-encoded voltage indicators in hiPSCs to monitor membrane potential changes in neurons and CMs (194). This can serve as an alternative to patch-clamp amplifier technology, which is beneficial for non-invasive high-throughput screening of hiPSC-derived-CMs for their electrophysiological characteristics. In addition, a gene-encoded ACh sensor (iAChSnFR) has been developed to monitor ACh release in hiPSCs-derived neurons (209). With the development of EMTA technology, Avet and colleagues have used BRET sensors to measure the activation of G proteins and β-arrestins in hiPSCs (12). The EMTA core biosensor consists of subdomains of the G protein effectors p63-RhoGEF, Rap1GAP, and PDZ-RhoGEF, which selectively interact with activated Gα_q/11_, Gα_i/o_, or Gα_12/13_, respectively. These structural domains are fused at their C-terminus with Renilla luciferase (RLucII) and co-expressed with different unmodified receptors and Gα heteromers. Upon GPCR activation, the energy donor fused effector is transferred to the membrane and binds to the activated Gα protein, bringing RLucII close to the energy acceptor Renilla green fluorescent protein, which is directed to the membrane through the CAAX motif (rGFP-CAAX), resulting in an increase in ebBRET. This technology does not overexpress or modify the native GPCR carried in the hiPSCs, preserving the original genetic information to the greatest extent, thus producing data of very high clinical significance.

The combination of hiPSCs and optical sensors can be used to study disease mechanisms and test personalized therapeutic drugs, particularly for diseases with genetic or molecular heterogeneity. By using hiPSCs and optical sensors, the underlying genetic and molecular mechanisms responsible for observing differences in drug response can be identified, leading to the development of more personalized and effective treatments. Furthermore, hiPSCs can be differentiated into somatic cell types, such as CMs and neurons, which are difficult to access in patients. By monitoring the functional properties of these cells in real-time using optical sensors changes in membrane potential, Ca^2+^ signaling, and neurotransmitter release, valuable additional insights into the pathophysiology of diseases can be obtained and utilized for developing future targeted therapeutics. Finally, the use of hiPSCs and optical sensors enables the monitoring of protein-protein interactions and dynamic studies using fusion proteins expressed at near physiological levels, providing a more accurate representation of the native cell environment compared to immortalized cell lines. Utilizing hiPSC will increase the clinical significance of the data describing patients’ specific GPCR function.

## Conclusion

Existing tools for characterizing GPCR distribution and interactions are abundant, and instruments with varying throughputs, frequencies, and signal intensities are available. The PCA- and RET-based characterization of GPCR interactions has been utilized frequently in immortalized cell lines, but infrequently in hiPSCs. If this is done out of concern for the complexity of hiPSC transfection, non-coding characterization can be used to characterize GPCR interactions. Currently, ligand- and antibody-mediated GPCR labeling and label-free DMR assays are the most practical methods to characterize GPCR localization and interactions after hiPSC stimulation. This does not require genetic labeling and is crucial for identifying GPCR signaling mechanisms and conducting more relevant drug screens. Because hiPSCs are patient-specific cell sources, they can be used to generate cardiac or neural tissue models for personalized drug screening platforms and to gain insight into patient-specific disease mechanisms. In addition, it has been demonstrated that hiPSCs can reproduce the phenotype of patients in various clinical states (210), including CM functionality, Ca^2+^ transient frequency, irregular heart rate, intracellular lipid levels, and lipid peroxidation (211). Therefore, it is necessary to expedite hiPSC-based GPCR targeting and interactions studies to develop personalized drugs for specific patient populations with specific individual targets, thereby enhancing therapeutic efficacy and minimizing adverse effects in clinical practice.

## Author contributions

This review was jointly completed by DO and GC. DO was responsible for guiding the writing and drawing, as well as revising the words and logic, grasping and designing the general direction of the paper. GC was responsible for writing the main body of the text, summarizing the table, and drawing the Figures. All authors contributed to the article and approved the submitted version.

## Glossary

**Table:**

ACh	Acetylcholine
AR	Adrenergic receptor
ACKR3	Atypical chemokine receptor 3
BiFC	Bimolecular Fluorescence Complementarity
BiLC	Bimolecular Luminescence Complementarity
BRET	Bioluminescence resonance energy transfer
BERKY	BRET biosensor with ER/K linker and YFP
GRK3ct	C-terminus of GPCR kinase 3
CMs	Cardiomyocytes
CCDs	Charge-coupled devices
CHO	Chinese hamster ovary
CMOS	Complementary metal-oxide semiconductor sensors
CFP	Cyan fluorescent protein
CyOFP1	Cyan-emitting fluorescent protein
cAMP	Cyclic adenosine monophosphate
DERET	Diffusion-Enhanced Resonance Energy Transfer
DSB	Double-stranded breaks
DMR	Dynamic mass redistribution
EMTA	Effector to plasma membrane translocation
EMCCDs	Electron-multiplied CCDs
EET	Electronic Energy Transfer
ESCs	Embryonic stem cells
ebBRET	Enhanced bystander BRET
EGFP	Enhanced green fluorescent protein
EYFP	Enhanced yellow fluorescent protein
FlAsH	Fluorescein-arsenic hairpin binder
FLIM	Fluorescence lifetime imaging microscopy
FRAP	Fluorescence recovery after photobleaching
FRET	Fluorescence resonance energy transfer
FDA	Food and Drug Administration
FRET	Förster Resonance Energy Transfer
GRK	G protein-coupled receptor kinase
GPCRs	G protein-coupled receptors
GEVIs	Gene-encoded voltage indicators
GCGR	Glucagon receptor
GLP-1	Glucagon-like peptide-1
GLP-1R	Glucagon-like peptide-1 receptor
GFP	Green fluorescent protein
H3R	Histamine H3 receptor
HDR	Homology-directed repair
HEK293	Human embryonic kidney-293
hiPSCs	Human induced pluripotent stem cells
iNC	Induced neuronal cell
IRES	Internal ribosome entry site
MMEJ	Microhomology-mediated end joining
NanoBiT	NanoLuc Binary Technology
NHEJ	Non-homologous end joining
UAA	Non-natural amino acid
OTR	Oxytocin receptor
PTH1R	Parathyroid hormone 1 receptor
PMTs	Photo-multiplier tubes
PET	Positron emission tomography
EP2	Prostaglandin E2 receptor
PCA	Protein Complementation Assay
PCA	Protein complementation assays
RLuc	Renillaluciferase
RET	Resonance energy transfer
TFFC	Three-fragment fluorescent complementation system
TR-FRET	Time-resolved FRET
TIRF	Total internal reflection fluorescence microscopes
TALEN	Transcription activator-like effector nucleases
YFP	Yellow fluorescent protein
